# Endogenous Nitric-Oxide Synthase Inhibitor ADMA after Acute Brain Injury

**DOI:** 10.3390/ijms15034088

**Published:** 2014-03-06

**Authors:** Carla S. Jung, Christian Wispel, Klaus Zweckberger, Christopher Beynon, Daniel Hertle, Oliver W. Sakowitz, Andreas W. Unterberg

**Affiliations:** Department of Neurosurgery, University of Heidelberg, Heidelberg D-69120, Germany; E-Mails: christianwispel@gmx.de (C.W.); klaus.zweckberger@med.uni-heidelberg.de (K.Z.); christopher.beynon@med.uni-heidelberg.de (C.B.); daniel.hertle@med.uni-heidelberg.de (D.H.); oliver.sakowitz@med.uni-heidelberg.de (O.W.S.); andreas.unterberg@med.uni-heidelberg.de (A.W.U.)

**Keywords:** asymmetric dimethylarginine, nitric oxide synthase, dimethylarginine dimethylaminohydrolases, protein-arginine methyltransferases, acute traumatic brain injury, controlled cortical impact

## Abstract

Previous results on nitric oxide (NO) metabolism after traumatic brain injury (TBI) show variations in NO availability and controversial effects of exogenous nitric oxide synthase (NOS)-inhibitors. Furthermore, elevated levels of the endogenous NOS inhibitor asymmetric dimethylarginine (ADMA) were reported in cerebro-spinal fluid (CSF) after traumatic subarachnoid hemorrhage (SAH). Therefore, we examined whether ADMA and the enzymes involved in NO- and ADMA-metabolism are expressed in brain tissue after TBI and if time-dependent changes occur. TBI was induced by controlled cortical impact injury (CCII) and neurological performance was monitored. Expression of NOS, ADMA, dimethylarginine dimethylaminohydrolases (DDAH) and protein-arginine methyltransferase 1 (PRMT1) was determined by immunostaining in different brain regions and at various time-points after CCII. ADMA and PRMT1 expression decreased in all animals after TBI compared to the control group, while DDAH1 and DDAH2 expression increased in comparison to controls. Furthermore, perilesionally ADMA is positively correlated with neuroscore performance, while DDAH1 and DDAH2 are negatively correlated. ADMA and its metabolizing enzymes show significant temporal changes after TBI and may be new targets in TBI treatment.

## 1. Introduction

Traumatic brain injury (TBI) triggers a cascade of changes inducing the development of secondary brain damage with nitric oxide (NO) playing an important role. NO is a strong vasodilator and regulates cerebrovascular tone and perfusion [1]. Therefore, it is not surprising that NO is one of the key players in the development of cerebral vasospasm after traumatic and aneurysmal subarachnoid hemorrhage (SAH) as well as in the development of secondary brain damage after severe TBI [2,3].

NO is synthesized from l-arginine by a family of NO-synthases (NOS): Two constitutive forms (cNOS) have been described, the endothelial NOS (eNOS) and the neuronal NOS (nNOS), as well as an inducible form (iNOS) [4]. The synthesis of NO via NOS can be inhibited by the endogenous competitive NOS-inhibitor asymmetric methylated l-arginine (ADMA), which is synthesized by protein-arginine methyltransferase 1 (PRMT1) and hydrolyzed by dimethylarginine dimethylaminohydrolase (DDAH1 and DDAH2) [5,6]. After TBI, an early decrease in intracerebral NO concentration occurs, which lasts from 30 min up to 6 h after TBI [2]. During this time, eNOS and nNOS activity appear normal and unchanged after TBI in humans [7]. The administration of exogenous NOS inhibitors leads to controversial effects [2,8–10]. Furthermore, administration of l-arginine resulted in reduced contusion volumes and had beneficial effects on cerebral blood flow [11–15]. Martens-Lobenhoffer *et al.* hinted at the endogenous NOS-inhibitor ADMA after TBI. They described an increase of ADMA levels in the cerebro-spinal fluid of patients with traumatic subarachnoid hemorrhage [16] parallel to the time-course of blood-brain barrier breakdown after TBI [17]. Variations in NO availability and controversial effects of exogenously administered NOS-inhibitors after TBI may be evoked by the competitive endogenous NOS inhibitor ADMA.

We, therefore, hypothesized that ADMA and the enzymes involved in ADMA-metabolism might be involved in TBI. Therefore, our goals were (1) to determine whether ADMA is expressed in brain tissue after TBI, focusing on the damaged brain site (TBI lesion) and the potentially influenceable penumbra (perilesional zone); (2) to establish whether time-dependent changes of ADMA expression exist; and (3) to investigate if the expression of enzymes involved in NO- and ADMA-synthesis might also be affected.

## 2. Results and Discussion

### 2.1. Neuroscore

Motor function performance after controlled cortical impact injury (CCII) is known to drop immediately after injury. In previous studies using a CCII rat model the impairment of motor function was found to be maximal during the first two days after injury [18–20]. We examined Garcia neuroscore performance [21] of control animals (*n* = 5) and of TBI animals at different time-points after CCII (*n* = 6; at each time point). Due to the experimental set-up no neurological testing could be performed within 30 min after CCII. All control animals reached the maximum attainable score of 18 points (100% level). In accordance with previous reports [18–20] we find a significant (*p* < 0.01) and immediate drop of 33% in performance over the first 2 days after TBI (13 ± 2 Garcia points; *n* = 24) compared to controls (18 Garcia points, *n* = 5).

### 2.2. ADMA, PRMT, DDAH and NOS Expression

**Figure 1.**
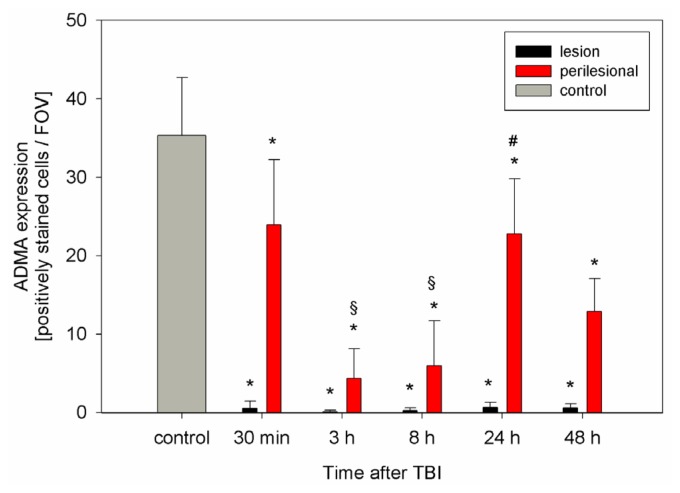
Expression of asymmetric dimethylarginine (ADMA) within the traumatic brain injury (TBI) lesion and perilesional area (sections of *n* = 5 animals at 30 min, 8 and 48 h, *n* = 6 at 3 and 24 h), as well as in control animals (*n* = 5), shown as mean of positively stained cells per examined fields of view (FOV) at a magnification of 400× (mean ± SD). * significant difference of lesional and perilesional ADMA expression in TBI animals compared with controls at any time-point after TBI (*p* < 0.01). Furthermore, significant changes of ADMA expression within the perilesional area are detectable: § A perilesional decrease in ADMA expression can be observed 3 and 8 h after TBI compared to 30 min after TBI (*p* < 0.001). # Thereafter, ADMA increases and peaks 24 h after TBI (*p* < 0.001 compared to 3 and 8 h after TBI, *p* > 0.05 compared to 30 min after TBI).

**Figure 2.**
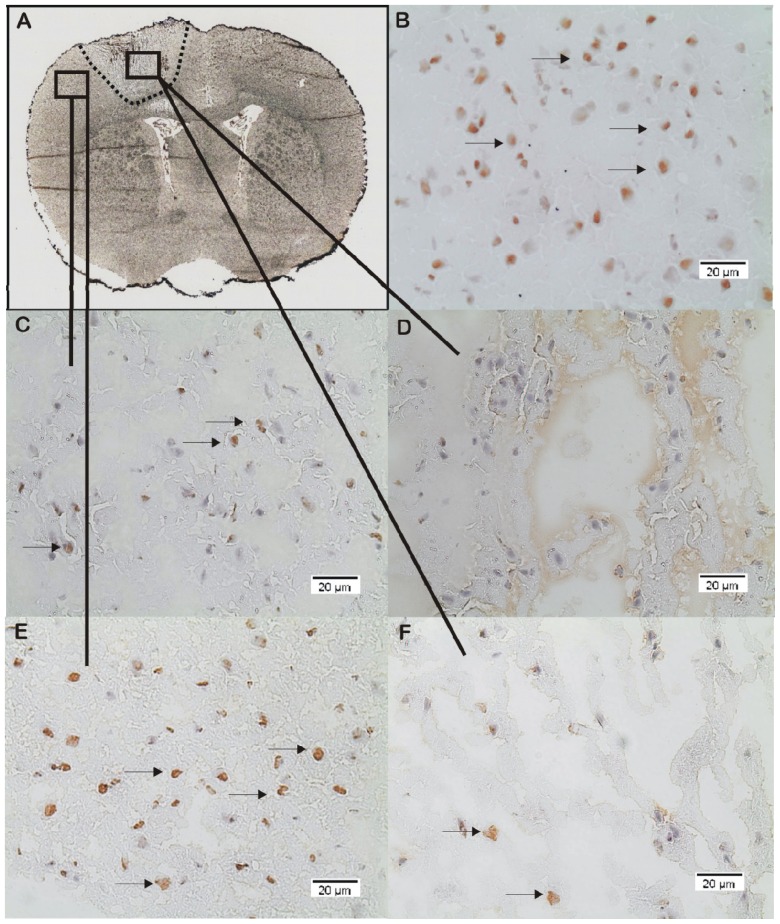
(**A**) Silver-staining and overview of rat brain cryosection 8 h after controlled cortical impact injury (CCII), indicating the TBI lesion (----: line). (**B**–**F**) Immunohistological staining and expression of ADMA (magnification: 200×); (**B**) ADMA expression in subcortical brain area of control animals, equivalent to the lesion and perilesional area of TBI animals; (**C**) ADMA expression within the perilesional area 8 h after CCII; (**D**) ADMA expression within the TBI lesion 8 h after CCII. No positively stained cells are detectable within this FOV. (**E**) and (**F**) ADMA expression 24 h after CCII; (**E**) The perilesional zone shows an increase in ADMA expression 24 h after CCII compared to 8 h after CCII; (**F**) ADMA within the TBI lesion 24 h after CCII. Only very few positively stained cells are detectable (→: examples for positively stained cells).

In control/sham animals which did not suffer from TBI, ADMA is strongly and evenly expressed in the supratentorial brain regions (35 ± 7 positively stained cells/FOV). After TBI ADMA expression decreases in all TBI animals compared to controls. However, significant differences can be found within the different brain regions (contusion/TBI lesion and penumbra/perilesional zone) (Figures 1 and 2). Thirty minutes after TBI, ADMA expression strongly decreases within the TBI lesion (*p* < 0.001) and remains low during the subsequent course while perilesional ADMA expression shows a decelerated decline (Figure 1). Early after TBI (30 min) the perilesional ADMA expression remains at 24 ± 8 positively stained cells/FOV. Then 3 to 8 h after TBI perilesional ADMA expression significantly decreases (*p* < 0.01) compared to the early phase (30 min), followed by an increase, reaching 24 h after TBI a second peak (*p* < 0.01) compared to the earlier values 3 and 8 h after TBI (Figures 1 and 2). Analyzing the time course of ADMA expression with respect to the Garcia neuroscore performance, we found a significant correlation in this perilesional area (cc = 0.46; *p* = 0.01; 95% CI: 0.12–0.7).

**Figure 3.**
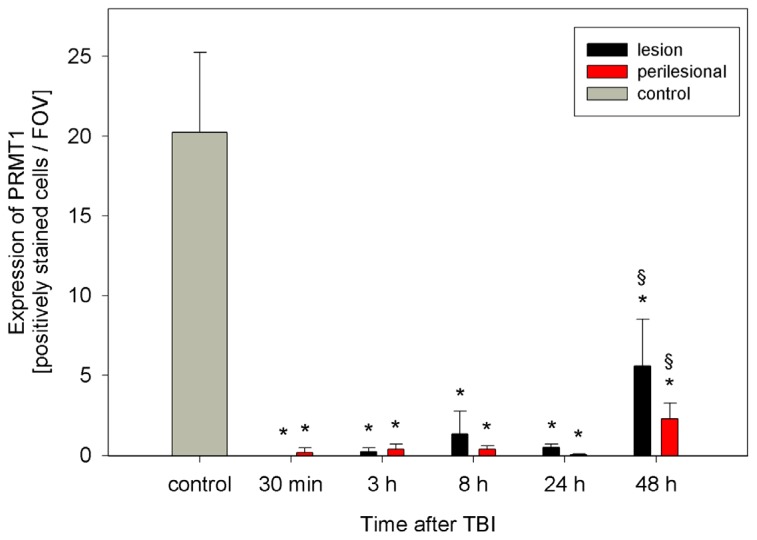
Expression of protein-arginine methyltransferase 1 (PRMT1) within the TBI lesion and perilesional area (sections of *n* = 6 animals at each time-point) as well as in control animals (*n* = 5), expressed as positively stained cells per field of view (FOV) at a magnification of 400× (mean ± SD). No positively stained cells can be detected within the lesion 30 min after TBI. * significantly decreased expression of ADMA in TBI animals compared to controls (*p* < 0.01). § significant increase (*p* < 0.001) of PRMT1 expression in the lesion and perilesional area compared to earlier time-points after TBI.

**Figure 4.**
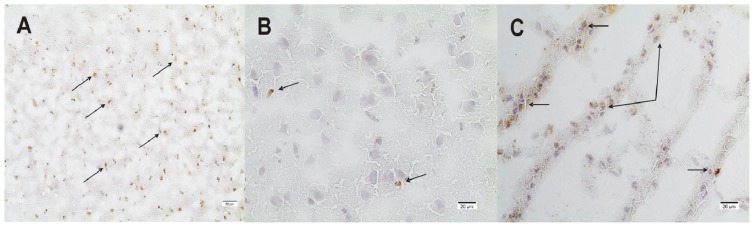
Immunohistological staining of PRMT1. (**A**) PRMT1 in control rat brain sections is evenly expressed (magnification 100×); (**B**) shows scattered PRMT1 positively stained cells 3 h after TBI within the perilesional zone and (**C**) 48 h after TBI within the lesion (**B**/**C**: magnification 200×) (→: examples for positively stained cells).

PRMT1, the enzyme which synthesizes ADMA by post-translational methylation of protein-bound L-arginine [22] can be detected in control animals (Figure 3). Control animals showed higher PRMT1 expression (20 ± 5 positively stained cells/FOV) than the animals submitted to TBI (range: 0–8 positively stained cells/FOV depending on the time-point after TBI) (*p* < 0.01). Early after TBI (30 min) PRMT1 expression disappears within the lesion. Then, three to eight hours later, some isolated scattered cells express PRMT1 (Figures 3 and 4). Forty-eight hours after TBI, PRMT1 expression increases especially within the lesion but also in the perilesional area compared to earlier time-points after TBI (*p* < 0.001) (Figure 3).

**Figure 5.**
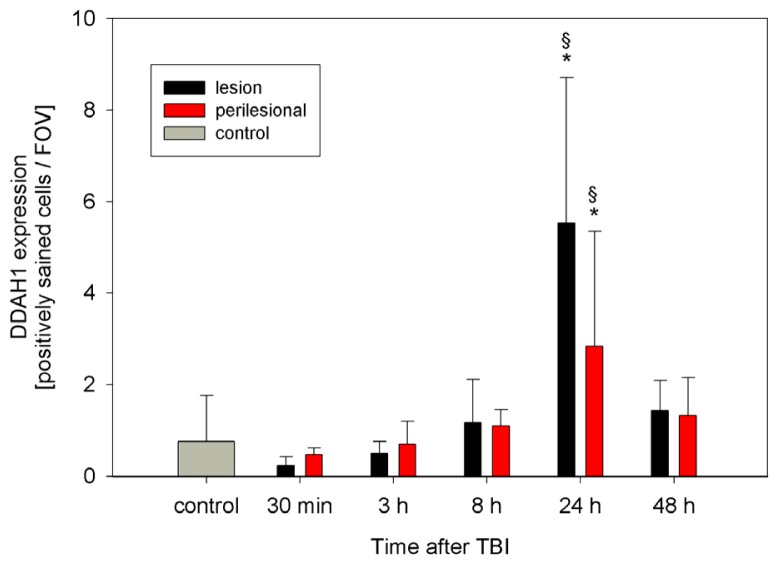
Expression of dimethylarginine dimethylaminohydrolases (DDAH)1 within the TBI lesion and perilesional area (sections of *n* = 6 at each time-point) as well as in control animals (*n* = 5), expressed as positively stained cells per field of view (FOV) at a magnification of 400× (mean ± SD). * significant difference in DDAH1 expression 24 h after TBI compared to controls. § significant increase of DDAH1 24 h after CCII in the lesion and perilesional area compared to 30 min and 3 h after CCII.

**Figure 6.**
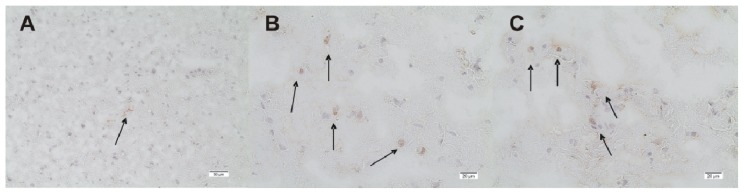
Immunohistological staining of DDAH1 (**A**–**C**). (**A**) DDAH1 in control rat brain sections (magnification 100×); (**B**) Perilesional DDAH1 expression shows positively stained cells 24 h after TBI and (**C**) lesional expression 24 h after TBI (**B**/**C**: magnification 200×) (→: examples for positively stained cells).

**Figure 7.**
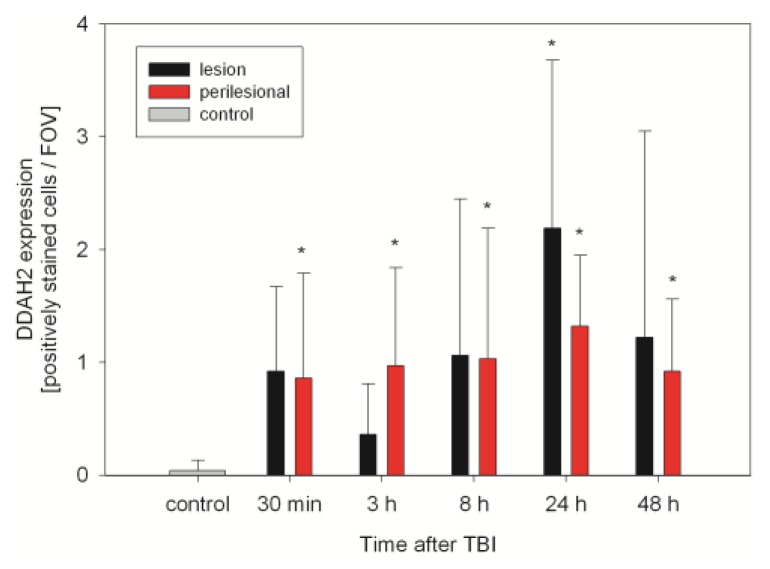
Expression of DDAH2 after CCII (sections of *n* = 6 animals at each time-point) within the TBI lesion and perilesional area as well as in control animals, expressed as positively stained cells per field of view (FOV) at a magnification of 400× (mean ± SD). * significant difference in DDAH2 expression compared to controls.

**Figure 8.**
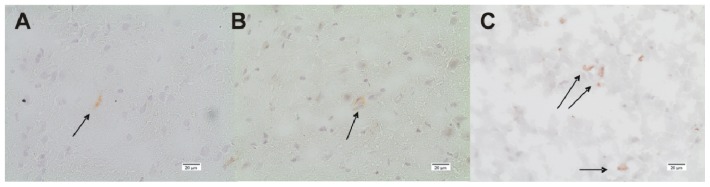
Immunohistological staining of DDAH2 (**A**–**C**). (**A**) DDAH2 expression in control rat brain sections is rare. In this FOV only one positively stained cell can be observed; (**B**) perilesional DDAH2 24 h after TBI and (**C**) lesional DDAH2 24 h after TBI (**A**–**C**: magnification 200×) (→: examples for positively stained cells).

**Figure 9.**
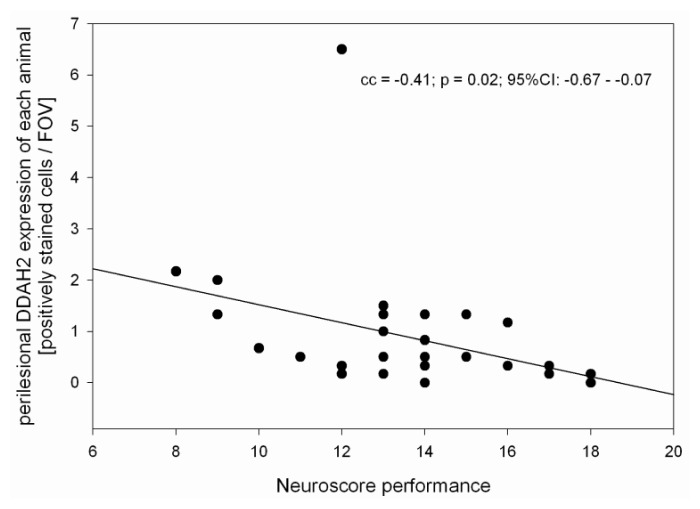
This scatter plot depicts individual expression of DDAH2 (positively stained cells/FOV) within the perilesional area of controls (*n* = 5) and of each TBI animal in accordance to their individual neuroscore performance before being sacrificed at the different time-points after TBI (*n* = 24; Animals 30 min after CCII were excluded because of missing neuroscores). Some dots are doubly assigned as three animals showed same neuroscore and DDAH2 expression values.

DDAH1 and DDAH2 are expressed in control and TBI animals. Especially in control animals and early after TBI, DDAH 1 expression is scattered and low (0.7 ± 1, range: 0–3 positively stained cells/FOV). Within 24 h after TBI, DDAH1 expression increases significantly inside the lesion and perilesionally to a peak (5.5 ± 3.1 and 2.8 ± 2.5 positively stained cells/FOV, *n* = 6, *p* < 0.01), which is also significantly higher than in the controls (*p* < 0.01) (Figures 5 and 6). DDAH2 expression after TBI is increased and stays elevated compared with controls (Figure 7). However, no significant changes can be observed within the lesion or perilesional area at the different time points (*p* > 0.05). DDAH expression is low (0 to 8 cells/FOV) and remarkably individual and variable resulting in strong standard-deviations at the different time-points after TBI (Figure 8). This might be related to the individual development of secondary brain injury and neuroscore performance. Corresponding to this, analyzing the individual DDAH expression of each animal at the different time-points with respect to the Garcia neuroscore performance (Figure 9), we found that in the perilesional area DDAH1 and DDAH2 expression were negatively correlated (cc = −0.42; *p* = 0.02; 95% CI: (−0.67)–(−0.8) and cc = −0.41; *p* = 0.02; 95% CI: (−0.67)–(−0.07), respectively).

**Figure 10.**
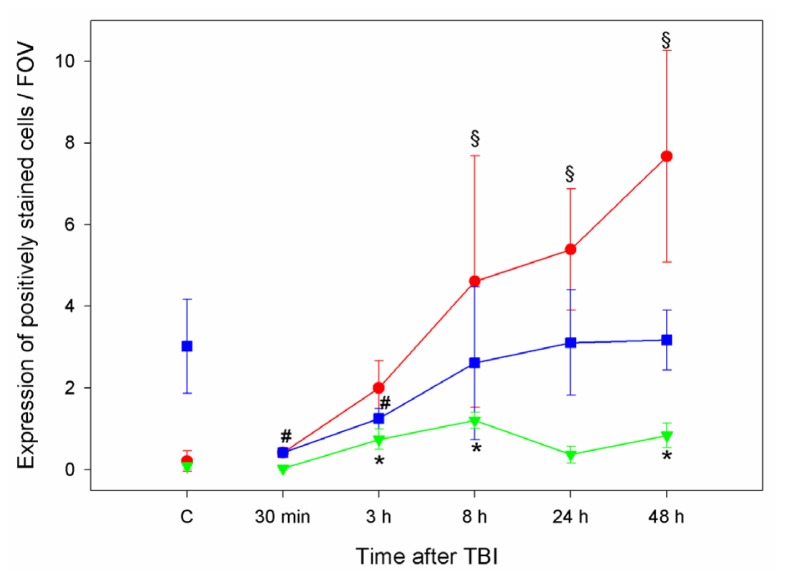
Line graph shows, iNOS (**red**), eNOS (**blue**) and NOS/ADMA ratio (**green**) expression in the perilesional zone at different time points after CCII as well as in controls (mean ± SD). § significant increase in iNOS expression. # significant decrease in eNOS expression 30 min and 3 h after TBI compared to controls as well as to 8, 24 and 48 h after TBI. * significant increase of NOS/ADMA ratio compared to controls.

In this study, a significant increase in iNOS expression, which starts 8 h after TBI can be observed (Figure 10). Comparable time-dependent and delayed increases in iNOS expression have been described previously [23–25]. eNOS expression significantly decreases 30 min to 3 h after TBI and subsequently regains control/sham levels 8 h after TBI (Figure 10). Comparable with these findings, Wada *et al.* reported after an immediate increase a sustained reduction in cNOS activity [24]. In humans, Gahm *et al.* could detect an iNOS peak 8 to 23 h after TBI, while eNOS and nNOS remained unchanged compared to controls [7]. Furthermore, Gahm *et al.* reported decreased nNOS expression after CCII in rats [23]. As iNOS generates and emits more NO than cNOS [26], iNOS derived NO has been suggested as a potential mediator of secondary brain injury [2,25]. NO by eNOS plays a major role in maintaining cerebral blood flow in the perilesional zone, which is on the one hand susceptible to secondary brain injury and on the other a potential target for treatment. Therefore, we focused on eNOS and iNOS expression.

As NO was not directly measured in these CCII animals, we calculated a ratio between NOS and ADMA, as indirect measure for NO availability: (eNOS + iNOS expression)/ADMA expression. In the perilesional area NOS/ADMA ratio significantly increases three hours after TBI (*p* < 0.01), which indicates, in accordance with previous reports [2,15,23,24], a delayed increase of NO in this region, which is likewise caused by an increase in iNOS expression [2,15,23,24] (Figure 10).

Reduced NO levels, as found early after TBI, in spite of unchanged or normal NOS activity can be explained if endogenous NOS inhibitors as ADMA play a role. Conversely, increased NO levels after TBI may be the result of increased NOS expression and reduced endogenous NOS inhibition, comparable to the observed delayed iNOS increase which was accompanied by an increase in DDAH and an overall decrease in ADMA compared to controls.

ADMA expression is detected in brain tissue of both controls and of animals which suffer TBI. Although ADMA expression strongly decreases within the traumatic lesion, it remains unchanged perilesionally during the early stage after TBI, followed by a decrease 3 h after TBI and a subsequent increase about 24 h after TBI. ADMA protein levels were not measured in this study. Martens-Lobenhoffer *et al.* described in a previous study increased ADMA protein levels in the cerebro-spinal fluid (CSF) and plasma of patients with traumatic subarachnoid hemorrhage after TBI [16]. Similarly increased ADMA levels accompanied by decreased NO levels in CSF and NOS inhibition have been demonstrated in cases of brain injury caused by aneurysmal subarachnoid hemorrhage [27,28]. Furthermore, Thampatty *et al.* reported elevated CSF ADMA level within 3 days after TBI in children [29]. The delayed detection of elevated ADMA levels in CSF and serum parallels the timescale for apoptosis and neuronal cell-death as well as the time-course of blood brain-barrier breakdown after TBI [17,30]. Therefore, the observed overall decrease in tissue ADMA expression, especially within the necrotic TBI lesion, compared to controls as well as the increase of ADMA expression within the perilesional zone 24 h after TBI in this study, which are also parallel to the described time-course of blood-brain barrier breakdown [17] do not seem to be conflicting. On the contrary, it underlines once more the variety of pathological and time-dependent changes observed after TBI. Furthermore, former experimental TBI studies in which NOS inhibitors were administered lead to controversial effects: In one study, which used the fluid percussion injury model, administration of l-NAME 5 min prior to injury resulted in an increased mortality rate [8]. In another study, administration of l-NAME pre- and post injury showed no adverse effects on outcome [31]. While some studies showed no effect, others demonstrated a reduction in edema, contusion volume and improvement in outcome [9,10,31]. The differing benefits achieved with administered NOS inhibitors become comprehensible when one considers the different time-points of administration together with the observed undulated course of ADMA and DDAH after TBI in our study. In addition, l-arginine, the substrate for NO production, also represents an ADMA competitor for NOS. In accordance with treatment successes reported on cerebral vasospasm after SAH [32,33], the administration of l-arginine resulted in a reduction of contusion volume after TBI [11–15]. l-arginine administration increased NO production [34] and, furthermore, it restored cerebral blood flow in various cortical contusion models of traumatic brain injury [12,13]. These observations suggest that l-arginine may overcome local competitive inhibition of NOS by ADMA after TBI and supports our hypothesis that ADMA is involved in the development of secondary brain damage, especially within the perilesional area surrounding the TBI lesion.

PRMT1, the enzyme which synthesizes ADMA by post-translational methylation of protein-bound l-arginine [22] can be detected in control animals, where it is evenly expressed in cortical neurons comparable with previous reports on mouse brain sections [35]. PRMT1 is significantly less expressed after TBI than in controls: Early after TBI (30 min) PRMT1 expression disappears within the lesion. Then, three to eight hours later, some isolated scattered cells express PRMT, reaching a maximum 48 h after TBI within the lesion but also in the perilesional area.

DDAH1 and DDAH2 hydrolyze ADMA. DDAH1 is mainly expressed in neuronal tissue [36,37] while DDAH2 is found perivascular similar to the eNOS expression [38]. In this study, however, no co-immunoprecipitation studies were performed. Both enzymes are expressed in control and TBI animals. Results on DDAH1 and DDAH2 in control brains are comparable to previous reports [39]. Within 24 h after TBI, DDAH1 expression increases significantly inside the lesion and perilesionally to a peak and is significantly higher than in the controls. DDAH2 expression after TBI is increased and stays elevated after TBI compared with controls. Furthermore, a negative correlation between DDAH1 and DDAH2 expression and neuroscore performance could be detected in the perilesional area.

Two different mechanisms may raise ADMA concentrations: (1) increased methylation of l-arginine by up-regulation of PRMT1 and (2) decreased hydrolysis of ADMA by DDAH. In hypercholesterolemia an up-regulation of PRMT mRNA expression has been reported in human endothelial cells by low-density lipoproteins (LDL) and suggested to be responsible for ADMA elevation [40]. While control animals showed strong PRMT1 expression, TBI resulted in reduced PRMT1 expression. We observed an early and strong decrease followed by an increase in PRMT1 expression within the injured hemisphere peaking 48 h after TBI. This was predominant within the lesion but also detectable in the perilesional area. The second mechanism, a decline in DDAH activity, occurs under pathological conditions such as cerebral vasospasm after subarachnoid hemorrhage and hypercholesterolemia [27,41]. After experimental subarachnoid hemorrhage DDAH2 expression was attenuated in cerebral vessels being affected by vasospasm [27]. In this traumatic brain injury study DDAH1 and DDAH2 expression significantly increased within the lesion and perilesionally, accompanied by a decrease in ADMA which could not be overcome by elevated PRMT1 expression 48 h after TBI. Therefore, both mechanisms may contribute to the overall effect.

Although we did detect a general decrease of ADMA after TBI, we cannot exclude local changes in ADMA expression with increased local levels of ADMA especially in cases of traumatic vasospasm after severe TBI. This is important, since distinct local and temporal profiles of cerebral blood volume may also contribute to secondary changes and functional outcome after TBI [2]. In the vulnerable perilesional area they seem to be potentially associated with the time course of sensory-motor deficit [42]. Similar, we found in our study a significant association between neuroscore and ADMA expression and a negative correlation of the neuroscore with DDAH1 and DDAH2 in the perilesional area.

DDAH inhibition and activation have been suggested as useful molecular probes to better understand cellular regulation of nitric oxide. Several potent DDAH inhibitors have recently been published [43] and suggested as potential therapeutic agents for treatment of pharmacological states associated with inappropriate under- and over-production of nitric oxide, such as septic shock [44]. Therefore, ADMA and especially DDAH changes in the perilesional area might offer potential new therapeutic targets in treatment of TBI and secondary brain damage development.

## 3. Experimental Section

### 3.1. Animals

Male Sprague-Dawley rats (*n* = 35) (Charles River, Germany) weighing 260 to 370 g were used in the present investigation. All experimental procedures were reviewed by the institutional committee for animal care and approved by the local veterinary authority (Regierungspräsidium Karlsruhe, Germany, G147/09).

### 3.2. Anesthesia and Brain Injury Model

Rats were anaesthetized in an isoflurane chamber (5%). Thereafter, anesthesia was maintained with a face mask using 1.5%–1.8% isoflurane, 30% O_2_, and 68% N_2_O. Via a catheter in the tail artery systemic blood pressure and blood gases were monitored. Body temperature was kept constant at 37.0 °C by a feedback controlled heating pad. After induction of anesthesia, the head was fixed in a stereotactic frame and a right-sided craniotomy was performed between the coronal, sagittal and lambdoid sutures with a micro-drill and under permanent cooling with saline. Specific attention was paid to leave the dura mater intact. Controlled cortical impact injury (CCII) was performed perpendicular to the surface of the brain as previously described [45]. The diameter of the impactor was 5 mm, the velocity 7.5 m/s, 1.5 mm depth of impression and the impact duration 300 ms. Craniotomy was closed immediately after CCII by replacing the bone-flap and fixation with dental cement.

### 3.3. Experimental Groups

One experimental group (*n* = 30) and one control group (*n* = 5) were investigated. All animals (experimental and control group) received craniotomy while rats of the control group were randomized to a sham group without receiving trauma/CCII. Animals in the experimental group were subjected to CCII and sacrificed after perfusion 30 min, 3, 8, 24 and 48 h after CCII (*n* = 6, at each time-point). Control animals were sacrificed directly after sham surgery.

### 3.4. Immunohistochemistry

After perfusion animal supratentorial brains intended for immunohistochemical staining were dissected out of the skull and snap frozen in 2-methylbutan (AppliChem, Darmstadt, Germany) and stored at −80°C for prompt cryosectioning. For immunostaining of different antibodies adjacent sections of 6 μm were used. On one object slide 2 brain sections with a minimum distance of 48 μm were mounted and both were stained with one of the following antibodies: Rabbit polyclonal antibody against endothelial NOS (eNOS), the inducible NOS (iNOS), ADMA and PRMT1 as well as goat polyclonal antibody against DDAH1 and DDAH2 were used with a 1:50 dilution for eNOS, iNOS, PRMT1, DDAH1 and DDAH2 (Abcam, Cambridge, UK). The antibody against ADMA was diluted 1:100. Sections without primary antibodies were used as negative controls in each staining set. Binding was visualized by a biotin conjugated secondary donkey immunoglobulin G antibody *versus* rabbit/goat immunoglobulin G antibody (Abcam, Cambridge, UK), adding Avidin/Biotin-complex (ABC) as well as 3,3′-Diaminobenzidine (DAB) as chromogen. Counterstaining was performed with hematoxylin. Distribution of immunohistologically stained cells within different brain areas (lesion, and perilesional zone) (e.g., Figure 2A) were analyzed at different time-points following CCII. In control animals the corresponding homotopic regions below the craniectomy were examined. As no difference could be found for any antibody staining among the different control regions their results were combined. Immunohistochemical staining was analyzed quantitatively by two independent observers who were blinded to the previous treatment protocols of animals: In two sections and in six fields of view (FOV) at a high-power magnification of 400×, the number of positively stained cells was counted and a mean value was calculated for each group and for the different brain areas at different time points (30 min, 3, 8, 24 and 48 h) following CCII. Furthermore, a ratio between expression of NO synthases and the NOS inhibitor ADMA was calculated as an indirect measure for NO availability, called in the following “NOS/ADMA ratio”: (eNOS + iNOS expression)/ADMA expression. For interobserver agreement, κ-statistics were performed, revealing excellent agreement (κ = 0.95).

### 3.5. Neurological Assessment

The neurological examination was performed referring to the modified grading system by Garcia *et al*. [21]. The Garcia test is a composite neurological test in which the rats are evaluated for various sensorimotor deficits: (1) spontaneous activity; (2) symmetry in four limb movement; (3) forepaw outstretching; (4) climbing; (5) body proprioception; and (6) response to vibrissal touch. For each mode of motor or behavioral deficit between 0 and 3 points were allocated, leading to a cumulative maximum score of 18 points as found in normal, healthy animals. Neurological deficits lead to lower scores (Figure 3) [21]. Garcia neuroscore performance was tested in all control animals (*n* = 5). TBI animals were examined before they were sacrificed at 3, 8, 24 and 48 h after CCII (*n* = 24; 6 animals at each time point). Due to the experimental set-up no neurological testing could be performed within 30 min after CCII (*n* = 6).

### 3.6. Statistics

Data are presented as mean value ± standard deviation (SD). A threshold value of significance (*p*-value) of less than 0.05 was applied. For statistical comparisons between groups, two-tailed Student’s *t*-tests for normally distributed data were used. Analysis of the normally distributed variables at the different time points was performed using one-way analysis of variance test (ANOVA) followed by multiple comparison post test (Tukey’s test and Dunnett’s test for comparison of multiple groups with one control group). Pearson’s correlation coefficient served to assess correlation. All calculations were performed with a standard statistical software package (GraphPad InStat, Version 3.05, GraphPad Software, Inc., La Jolla, CA, USA).

## 4. Conclusions

This is, to the best of our knowledge, the first study that describes temporal profiles of NOS, ADMA, PRMT1, DDAH1 and DDAH2 in the brain after experimental contusion/TBI. The main findings of our experiments are: (1) ADMA is expressed in brain sections of animals after suffering TBI as well as in controls; (2) TBI changes ADMA expression strongly: ADMA expression decreased in all time-groups after TBI compared to the control group. However, within the lesion ADMA expression drops immediately and stays low, while the level in the perilesional area decreases 3 h after TBI and increases 24 h after TBI compared to previous time-groups, but remains at all times lower than in controls; (3) PRMT1 also decreased in all animals after TBI compared to the control group, while; (4) DDAH1, and DDAH2 increased in comparison to controls; (5) In the perilesional area ADMA is positively correlated with neuroscore performance; and (6) DDAH1 and DDAH2 are negatively correlated with neuroscore performance.

Although, ADMA and the enzymes involved in ADMA-metabolism show significant changes after TBI, future experiments have to prove if the competitive endogenous NOS inhibitor ADMA effects variations in NO availability and previously described controversial effects of exogenously administered NOS-inhibitors after TBI. Nevertheless, ADMA and its metabolizing enzymes might be new targets in TBI treatment.
